# Supplementary material for: Calretinin-expressing islet cells: a source of pre- and post-synaptic inhibition of non-peptidergic nociceptor input to the mouse spinal cord
Source: bioRxiv. 2023 Jun 5:2023.06.01.543241. Preprint. [Version 1] doi: 10.1101/2023.06.01.543241 (PMC10274676; doi:10.1101/2023.06.01.543241)
Supplement: Supplement 1 [file media-1.pdf]

Calretinin-expressing islet cells: a source of pre- and post-synaptic inhibition of non-peptidergic nociceptor input to the mouse spinal cord

Olivia C. Davis[1,7], Allen C. Dickie[1,7], Marami B. Mustapa[1,6], Kieran A. Boyle[1], Tyler J. Browne[2], Mark A. Gradwell[2], Kelly M. Smith[2], Erika Polgár[1], Andrew M. Bell[1], Éva Kókai[1], Masahiko Watanabe[3], Hendrik Wildner[4], Hanns Ulrich Zeilhofer[4], David D. Ginty[5], Robert J. Callister[2], Brett A. Graham[2*], Andrew J. Todd[1*] and David I. Hughes[1*]

[1]School of Psychology and Neuroscience, Sir James Black Building, University of Glasgow, Glasgow, G12 8QQ, UK; [2]School of Biomedical Sciences and Pharmacy, Faculty of Health and Medicine, University of Newcastle, Callaghan, NSW, Australia; [3]Department of Anatomy, Hokkaido University School of Medicine, Sapporo 060-8638, Japan; [4]Institute of Pharmacology and Toxicology, University of Zurich, 8057 Zürich, Switzerland; [5]Department of Neurobiology, Howard Hughes Medical Institute, Harvard Medical School, 220 Longwood Avenue, Boston, MA 02115, USA; [6]Present address: Faculty of Medicine and Defence Health, National Defence University of Malaysia, 57000, Kuala Lumpur, Malaysia

[7]These authors contributed equally: Olivia Davis and Allen Dickie

Number of words in abstract: 170
main text: 6591
online methods: 3423
Number of references: 94
Number of figures: 12 (plus 4 supplementary)

*Corresponding authors:

David I. Hughes, Spinal Cord Group, Sir James Black Building, University of Glasgow, Glasgow, G12 8QQ, UK. Email: David.I.Hughes@glasgow.ac.uk

Andrew J. Todd, Spinal Cord Group, Sir James Black Building, University of Glasgow, Glasgow, G12 8QQ, UK. Email: Andrew.Todd@glasgow.ac.uk

Brett A. Graham, School of Biomedical Sciences and Pharmacy, Faculty of Health and Medicine, University of Newcastle, Callaghan, NSW, Australia. Email: Brett.Graham@newcastle.edu.au

Fig S1

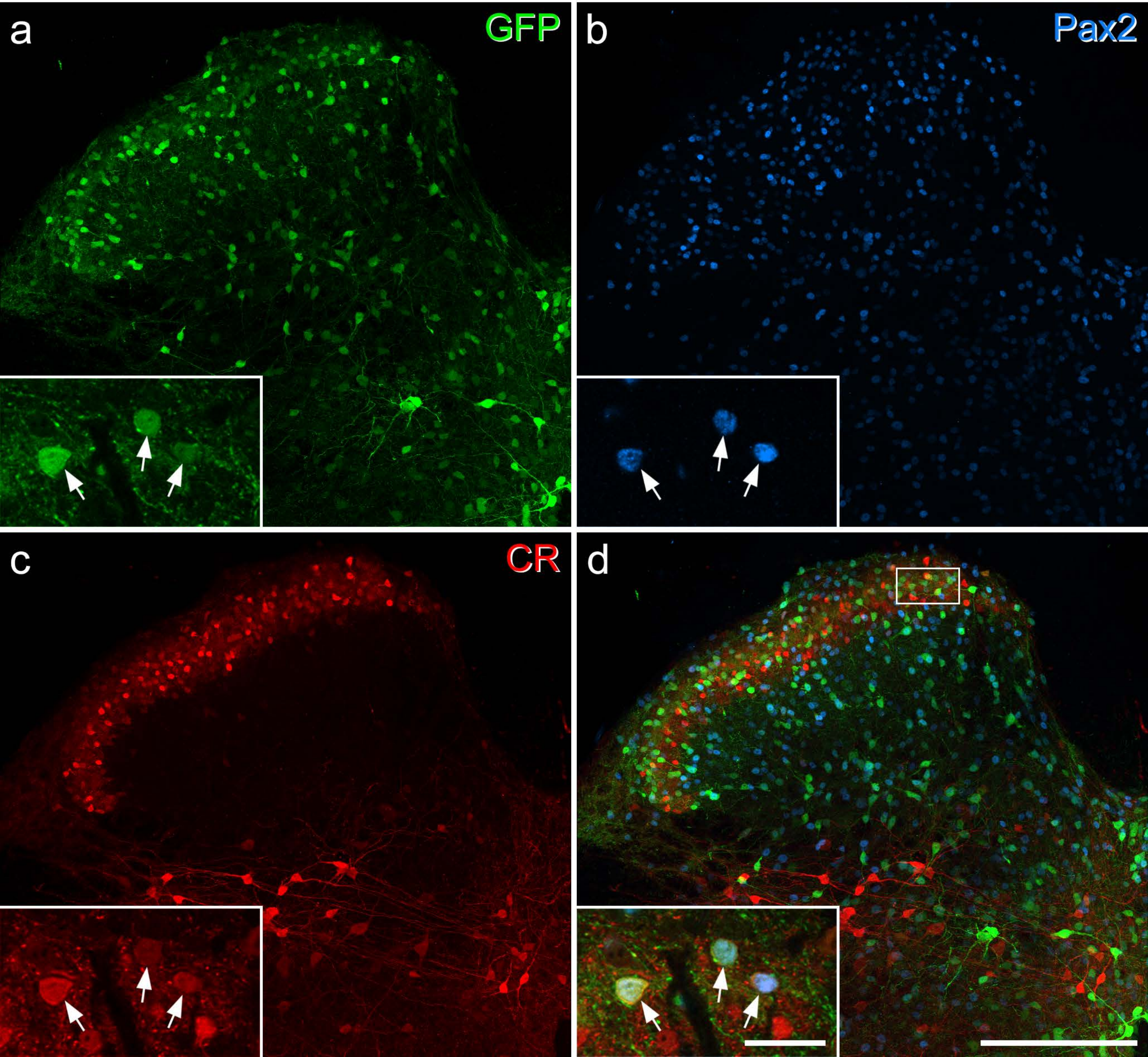

Fig S2

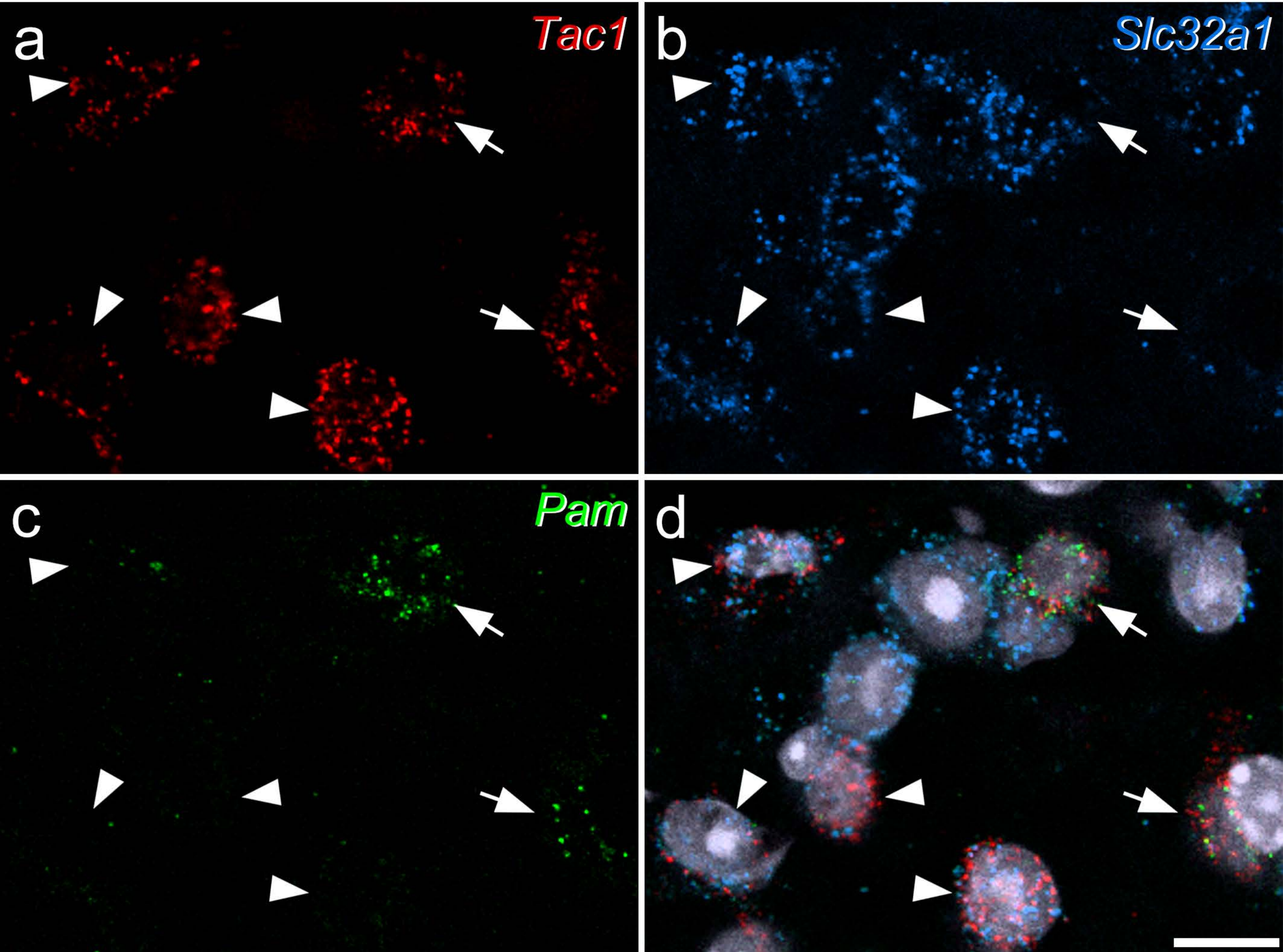

Fig S3

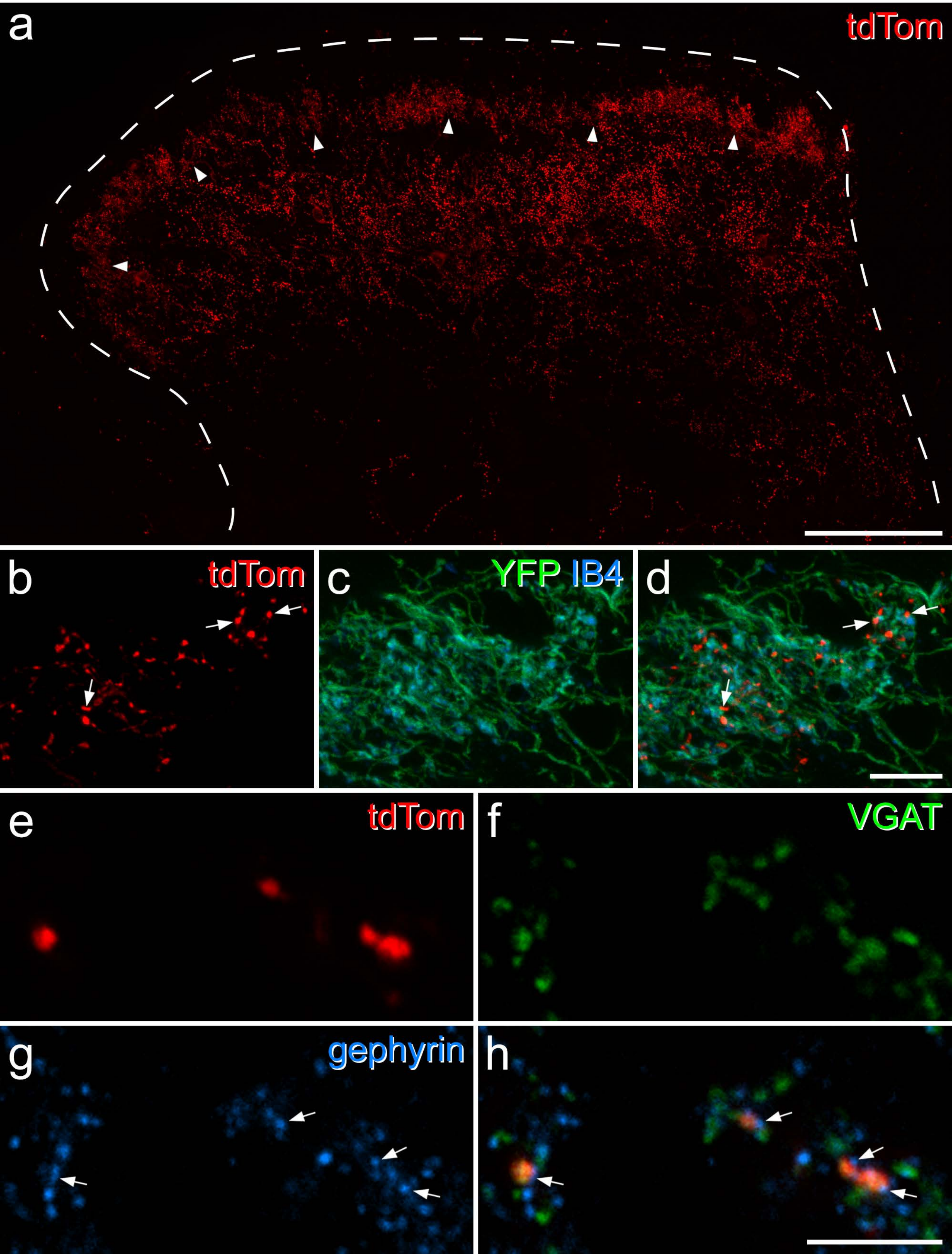

Fig S4

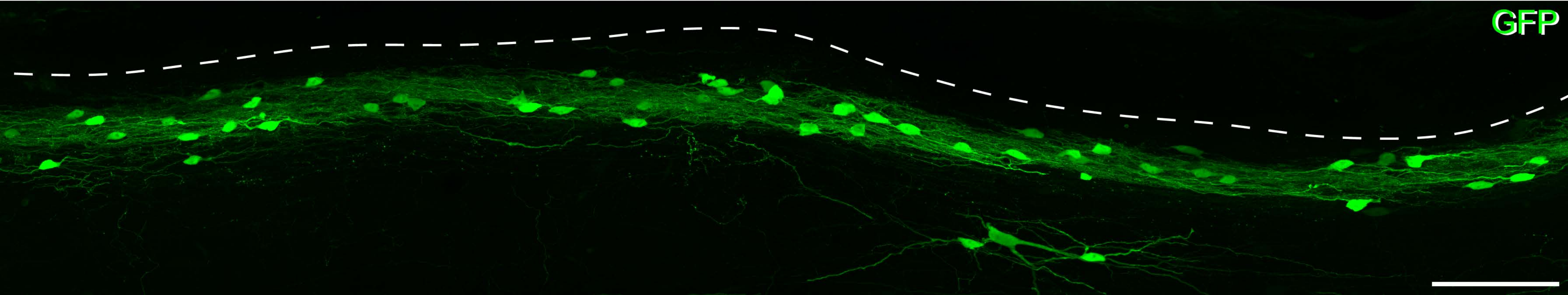

Table S1 Mouse lines used in the study

	Source	Reference	Catalogue number, RRID
CR::GFP	H Monyer	Caputi et al., 2009	
Pnoc::GFP	HU Zeilhofer	Smith et al., 2020	
$Rorb^{CreERT2}$	DD Ginty	Abraira et al., 2017	
CR^{Cre}	Jackson Laboratory		JAX:010774<br>RRID:IMSR_JAX:010774
$MrgD^{ChR2\text{-}YFP}$	Mutant Mouse Resource & Research Centers		036112-UNC<br>RRID:MMRRC_036112-UNC
$VGAT^{Flp}$	Jackson Laboratory		JAX:029591<br>RRID:IMSR_JAX:029591
Ai9	Jackson Laboratory		JAX: 007909<br>RRID:IMSR_JAX:007909
Ai32	Jackson Laboratory		JAX:012569<br>RRID:IMSR_JAX:012569
Ai34	Jackson Laboratory		JAX:012570<br>RRID:IMSR_JAX:012570

Table S2 Antibodies used in the study

**Antigen**	**Host species**	**Source**	**Catalogue no**	**Dilution**	**RRID**
mCherry*	Rat	Invitrogen	M11217	1:1000	RRID:AB_2536611
mCherry*	Rabbit	Abcam	Ab167453	1:1000	RRID:AB_2571870
GFP**	Chicken	Abcam	ab13970	1:1000	RRID:AB_300798
GFP†	Rabbit	M Watanabe		1:10,000	RRID:AB_2571573
Pax2	Rabbit	Life Technologies	716000	1:1000	RRID:AB_2533990
Calretinin	Goat	Swant	CG1	1:1000	RRID:AB_10000342
Homer	Goat	M Watanabe		1:1000	RRID:AB_2631104
PAP	Chicken	Aves	PAP	1:1000	RRID:AB_2313557
Somatostatin	Guinea pig	P Ciofi		1:1000	
Gephyrin	Mouse	Synaptic Systems	147 021	1:1000	RRID:AB_2232546
VGAT	Goat	M Watanabe		1:1000	RRID:AB_2571623

*The mCherry antibody also recognises tdTomato

**The GFP antibody also recognises YFP. The chicken anti-GFP antibody was used in all cases in which YFP was to be revealed with immunofluorescence.

†The anti-rabbit GFP antibody was used for immunoperoxidase labelling in experiments involving electron microscopy.

Table S3 RNAscope probes used in the study

**Probe**	**Protein/peptide**	**Channel numbers**	**Catalogue numbers**
tdTomato	tdTomato	2	317041
Tac1	Preprotachykinin 1	3	410351
Gad1	GAD67	1	400951
Pam	Peptidyl-glycine alpha-amidating monooxygenase	2	457271
Slc32a1	Vesicular GABA transporter	3	319191
RNAscope multiplex positive control (*Polr2a*, *Ppib*, *Ubc*)	Polr2a: DNA-directed RNA polymerase II subunit RPB1; Ppib: Peptidyl-prolyl cis-trans isomerase B; Ubc: Polyubiquitin-C	1,2,3	320881
RNAscope multiplex negative control (*dapB*)	dapB: 4-hydroxy-tetrahydrodipicolinate reductase (derived from B Subtilis)	1,2,3	320871

Supplementary figure legends

Fig S1 Labelling of iCRs in the Pnoc::GFP mouse. **a**-**c**: a transverse section from one of these mice has been reacted to reveal GFP (green), Pax2 (blue) and calretinin (CR, red). **d**: a merged image. The inset images show 3 GFP-labelled cells (arrows), which are immunoreactive for both Pax2 and calretinin. The box in **d** shows the area from which the inset was taken. Main images are maximum intensity projections of 15 confocal optical sections at 2 μm z-spacing. The inset images are from a single optical section. Scale bars = 200 μm (main images) and 25 μm (insets).

Fig S2 Fluorescent *in situ* hybridisation for *Tac1*, *Slc32a1*, and *Pam* mRNAs in lamina II of a wild-type mouse. *Slc32a1* encodes the vesicular GABA transporter, and is present in inhibitory interneurons, while *Pam* codes for peptidyl-glycine alpha-amidating monooxygenase, the enzyme that amidates substance P. **a**-**c** show individual channels, while **d** is a merged image that also includes nuclear staining with NucBlue (grey). This field contains several *Tac1*-containing cells, four of which also contain *Slc32a1* and are therefore inhibitory (arrowheads). Two excitatory *Tac1*-containing cells (identified by lack of *Slc32a1*) are marked with arrows. The density of transcripts for *Pam* is much lower in the four inhibitory Tac1 cells than in the two excitatory cells. Images are maximum intensity projections of confocal optical sections (1 μm z-separation) through the full thickness of the section. Scale bar = 10 μm.

Fig S3 Axons of Rorb cells revealed by staining for tdTomato (tdTom) in the Rorb^{CreERT2};Ai34 cross. **a**: a scan through the dorsal horn of a Rorb^{CreERT2};Ai34;MrgD$^{ChR2\text{-}YFP}$ mouse reveals a dense band of tdTomato labelling in lamina II (marked by arrowheads), as well as

staining in deeper laminae (III-IV). There is a partial separation between these two layers. **b**-**d**: a section from the same cross scanned through lamina II to reveal tdTom (red), YFP (green) and IB4 binding (blue). The tdTomato-labelled boutons in lamina II are closely associated with MrgD axonal boutons, revealed by IB4 binding in YFP-labelled varicosities. Arrows point to appositions between individual tdTomato-labelled and MrgD+/IB4+ boutons. **e**-**h**: a scan from lamina II of a $Rorb^{CreERT2}$;Ai34 mouse, immunostained to reveal tdTom (red), VGAT (green) and gephyrin (blue). Four tdTomato-labelled boutons are present in this field, and all are VGAT-immunoreactive. Each of these is in contact with a gephyrin punctum (arrows in **g** and **h**). The image in **a** is a projection of 27 optical sections at 1 μm z-separation, those in **b**-**d** are projections of 11 optical sections at 0.3 μm z-separation, while those in **e**-**h** are from a single optical section. Scale bars: 100 μm (**a**), 10 μm (**b**-**d**) and 5 μm (**e**-**h**).

Fig S4 Intersectional targeting of inhibitory Tac1 cells. A sagittal section through the region of the injection site in a $Tac1^{Cre}$;$VGAT^{Flp}$ mouse that had received an injection of AAV.C^{on}/F^{on}.GFP, immunostained to reveal GFP. The dashed line represents the dorsal surface of the grey matter. Note that the great majority of GFP labelled cells are located in lamina II, and that their processes remain in this lamina. The image is a projection of 27 confocal optical sections at 1 μm z-spacing. Scale bar = 100 μm.
